# The Glutamine-α-Ketoglutarate Metabolic Axis Controls Vascular Smooth Muscle Cell Function

**DOI:** 10.3390/cells15030230

**Published:** 2026-01-26

**Authors:** Kelly J. Peyton, Xiao-Ming Liu, Giovanna L. Durante, William Durante

**Affiliations:** Department of Medical Pharmacology and Physiology, University of Missouri, Columbia, MO 65212, USA

**Keywords:** aminotransferase, collagen, glutaminase, glutamine, α-ketoglutarate, migration, proliferation, vascular smooth muscle cells, viability

## Abstract

Glutamine is a known regulator of vascular smooth muscle cell (VSMC) function, but the molecular pathways underlying this response remain incompletely understood. This study investigated how glutamine metabolism influences VSMC behavior and identified the responsible enzymes and metabolites. Glutamine deprivation markedly reduced VSMC proliferation, migration, and collagen synthesis, while modestly decreasing viability. Pharmacological inhibition of glutaminase-1 (GLS1) or aminotransferases (AT) similarly suppressed these cellular functions, whereas inhibiting glutamate dehydrogenase 1 (GLUD1) had no effect. Metabolite analysis revealed that glutamine deprivation or AT inhibition, but not GLUD1 inhibition, reduced intracellular α-ketoglutarate (αKG) concentrations, establishing AT as the primary enzyme converting glutamine-derived glutamate to αKG. To identify which metabolite drives VSMC responses, glutamine-starved cells were supplemented with various glutamine-derived molecules. The cell-permeable αKG analog dimethyl-αKG significantly restored VSMC proliferation, migration, collagen synthesis, and survival, while ammonia only enhanced viability, demonstrating αKG’s primary role in mediating glutamine-dependent functions. These findings establish that glutamine metabolism via the GLS1-AT-αKG pathway is a critical driver of VSMC activation and survival. Targeting this glutamine-αKG metabolic axis through GLS1 inhibition, AT blockade, or downstream αKG disruption offers a compelling therapeutic strategy for ameliorating fibroproliferative vascular diseases, including atherosclerosis, post-angioplasty restenosis, and pulmonary hypertension.

## 1. Introduction

Glutamine is the most abundant amino acid in the circulation and plays a versatile role in cell metabolism [1,2,3]. Upon entering the cell, glutamine is used in the cytosol to produce purines, pyrimidines, nucleotides, asparagine, and glucosamine. However, the predominant fate of glutamine involves its mitochondrial metabolism through glutaminolysis, where it is converted to glutamate and ammonia (NH_3_) by the rate-limiting enzyme glutaminase (GLS) [4]. Two distinct isoforms of GLS exist, GLS1 and GLS2, which display unique structural and kinetic properties and tissue distribution. While GLS2 expression is localized to the liver, where it supplies nitrogen for the urea cycle, GLS1 is expressed in most cells outside the liver, including vascular smooth muscle cells (VSMCs) [5,6].

Following glutamine deamidation, mitochondrial glutamate is subsequently converted into α-ketoglutarate (αKG) by glutamate dehydrogenase 1 (GLUD1) or by several mitochondrial aminotransferases, including glutamic-pyruvic transaminase 2 (GPT2) and glutamic-oxaloacetic transaminase 2 (GOT2) [7]. The GLUD1-catalyzed reaction is particularly notable as it represents an anaplerotic pathway that simultaneously generates nicotinamide adenine dinucleotide (NADH) and NH_3_ as byproducts, whereas the aminotransferase routes couple αKG production to the synthesis of alanine (via GPT2) or aspartate (via GOT2), thereby linking glutamine metabolism to biosynthetic networks [1,2,3]. Mitochondrial αKG then enters the tricarboxylic acid (TCA) cycle, where it can yield adenosine triphosphate (ATP) via oxidative phosphorylation, citrate that can be exported for lipid and sterol synthesis, and the reducing equivalents NADH and the reduced form of flavin adenine dinucleotide (FADH_2_) that sustain redox homeostasis and drive ATP synthesis [4]. Alternatively, glutamate can be transported from the mitochondria to the cytosol, where it contributes to the synthesis of glutathione and non-essential amino acids (including proline, arginine, and ornithine) and facilitates the import of cystine through the cystine–glutamate antiporter system [1,2]. Similarly, the export of αKG from the mitochondria via solute transporters stimulates mammalian target of rapamycin complex 1 (mTORC1) activity through mechanisms involving αKG-dependent dioxygenases and influences the epigenetic landscape through αKG-dependent histone and DNA demethylases [4]. Through these diverse and interconnected metabolic fates, glutamine fulfills both the energetic and macromolecular biosynthetic requirements of cells and plays a pivotal role in regulating key cellular processes, including proliferation, survival, migration, activation, and differentiation [8,9,10,11,12,13].

Given its central role in cellular metabolism, dysregulated glutamine catabolism has emerged as a common feature across multiple disease states. A growing number of studies indicate that reprogramming of glutamine metabolism underlies the pathogenesis of many diseases, most notably cancer but also neurological, respiratory and liver disease, infectious and renal disease, and mitochondrial disorders [8,14,15,16,17,18,19]. Of relevance to cardiovascular health, altered glutaminolysis is a central feature of several vascular and cardiac disorders. Early investigations identified increased GLS1 expression in pulmonary arterioles of patients with pulmonary arterial hypertension and in rodent and simian models of pulmonary hypertension [6]. Importantly, pharmacological inhibition of GLS1 improved arterial remodeling and ameliorated disease progression in animal models of pulmonary hypertension [20]. Building on these findings, subsequent studies demonstrated that GLS1 dysregulation participates in the etiology of arterial stiffness, heart failure, calcific aortic valve disease, and arterial calcification [21,22,23,24]. More recently, disturbances in glutamine metabolism were demonstrated to influence arterial remodeling in atherosclerosis and following arterial injury, further highlighting the broad implications of glutaminolysis in vascular pathobiology [25,26,27].

A key driver of vascular disease pathology is the phenotypic plasticity of vascular smooth muscle cells (VSMCs), which can transition between various phenotypic states that promote disease development [28]. Accumulating evidence demonstrates that glutamine plays a fundamental role in regulating VSMC phenotype and function. Specifically, glutamine induces VSMC phenotype switching from a quiescent, contractile state to a proliferative, synthetic state [29]. Exogenous administration of glutamine enhances VSMC growth and migration, while intracellular glutamine levels determine VSMC-related thrombogenicity [29,30,31]. Furthermore, glutaminolysis increases VSMC proliferation and migration and neointimal thickening following arterial injury [27,32]. Conversely, blocking glutamine transport suppresses VSMC growth, movement, and ligation-induced neointima formation in mice [29,33].

While these studies clearly establish a key role for glutamine in regulating VSMC function, critical knowledge gaps remain. The enzymatic pathways within glutaminolysis that mediate glutamine’s cellular actions have not been definitively identified. In addition, the downstream metabolites of glutamine that are responsible for the observed effects on VSMC function remain unknown. Moreover, whether glutaminolysis regulates additional VSMC processes beyond differentiation, proliferation, and migration has not been thoroughly investigated. Accordingly, the current study was designed to systematically dissect the role of glutaminolysis in VSMC function. Specifically, we determined the importance of the enzymes involved in glutaminolysis, GLS1, GLUD1, and aminotransferases, in mediating the actions of glutamine on VSMC function. Furthermore, we investigated the ability of various downstream metabolites of glutamine to regulate VSMC properties. Finally, this study also determined whether glutaminolysis regulates VSMC viability and collagen synthesis, key processes involved in vascular remodeling and disease. Collectively, these investigations provide mechanistic insights into how glutamine metabolism regulates VSMC function and identify specific enzymatic nodes that may serve as therapeutic targets in ameliorating vascular disease.

## 2. Materials and Methods

### 2.1. Reagents

Minimum essential medium, ethylenediaminetetraacetic acid (EDTA), β-aminopropionitrile, trypsin, crystal violet, penicillin, streptomycin, *N*-ethylmaleimide, bovine serum albumin, elastase, collagenase, dialyzed bovine calf serum, sodium dodecyl sulfate (SDS), NaOH, trichloroacetic acid (TCA), phosphate-buffered saline (PBS), acetic acid, glutamine, glutamate, aspartic acid, asparagine, dimethyl-αKG, ammonium chloride, bromophenol blue were from Sigma-Aldrich (St. Louis, MO, USA). Rainbow molecular weight markers were from GE Healthcare (Piscataway, NJ, USA). Telaglenastat (CB-839), bis-2-(5-phenylacetamido-1,3,4-thiodiazol-2-yl)ethyl sulfide (BPTES), R162, and aminooxyacetic acid (AOA) were from Selleckchem (Houston, TX, USA). The antibody against GLS1 was from Abcam (Cambridge, MA, USA), while the antibody against β-actin was from Santa Cruz Biotechnology (Santa Cruz, CA, USA). [^3^H]Thymidine (20 Ci/mmol) and [^3^H]proline (100 Ci/mmol) were from Perkin Elmer (Boston, MA, USA).

### 2.2. Cell Culture

Primary VSMCs were isolated from rat aorta, characterized, and propagated, as we have previously reported [34,35,36]. VSMCs were cultured in minimum essential medium containing Earle’s salts, 10% bovine calf serum, 100 U/mL penicillin, and 100 U/mL streptomycin. Human aortic VSMCs were purchased from Cell Biologics (Chicago, IL, USA) and maintained in basal medium with insulin, fibroblast growth factor, epidermal growth factor, penicillin, hydrocortisone, streptomycin, and amphotericin B, with 10% fetal bovine serum. Cultures were incubated in a humidified atmosphere of 95% air and 5% CO_2_ at 37 °C. Unless otherwise indicated, culture medium contained 2 mM L-glutamine. Prior to experimental treatments, VSMCs were growth-arrested by incubation of rat VSMCs in serum-free medium supplemented with bovine serum albumin (1%) and human VSMCs in basal medium without growth supplements for 48 h.

### 2.3. Cell Proliferation and DNA Synthesis

VSMC proliferation was assessed by direct cell counting and measuring DNA synthesis [35,36,37]. Cells were seeded (1–2 × 10^4^ cells/well) onto 12-well plates in serum-containing media. Following synchronization by serum or growth supplement starvation, cells were treated with complete medium in the absence or presence of various test compounds. During the treatment interval, medium was replaced every 48 h with fresh serum- or growth supplement-replete medium. Following treatment, cells were harvested at indicated time points using trypsin (0.5%)-EDTA (53 mM) and counted using an automated cell counter (Moxi Z, ORFLO Technologies, Ketchum, ID, USA). DNA synthesis was quantified by measuring the incorporation of [^3^H]thymidine into newly synthesized DNA. VSMCs were pulsed with [^3^H]thymidine (1 µCi/mL) at 37 °C for four hours. Following the labeling period, cells were then washed three times with ice-cold PBS to remove unincorporated [^3^H]thymidine and soluble nucleotide pools. Cells were then fixed with ice-cold TCA (10%) to further remove unincorporated radioactivity, followed by two additional washes to remove residual TCA. The TCA-precipitated DNA was then solubilized with SDS (0.2%)/NaOH (0.2 N) for two hours at room temperature with gentle agitation. Solubilized samples were transferred to scintillation vials and radioactivity was quantified by scintillation counting (Tricarb liquid scintillation analyzer, model 2100, Packard, Meriden, CT, USA), as previously noted [35,37].

### 2.4. Cell Migration

VSMC migration was monitored using a transwell chamber assay based on the modified Boyden chamber principle. Cells were seeded (4 × 10^4^ cells) onto the upper chamber of transwell inserts containing polycarbonate membranes (8.0 µm) (Corning Incorporated, Corning, NY, USA). The upper chamber contained serum-free medium with experimental treatments, while the lower chamber was filled with complete culture medium containing 10% serum as a chemoattractant. Following 24 h of incubation at 37 °C, non-migrated cells remaining on the upper surface of the membrane were removed by gentle swabbing with a cotton-tipped applicator moistened with PBS. Migrated cells adhering to the lower surface of the membrane were fixed and stained with 0.5% (*w*/*v*) crystal violet for 30 min at room temperature. Inserts were then washed three times with PBS to remove excess stain and air-dried. Bound crystal violet was solubilized by adding 33% (*v*/*v*) acetic acid to each insert and incubating on an orbital shaker for 10 min at room temperature. The resulting eluant was collected and absorbance was measured at 590 nm using a plate reader. Migration was quantified as a percentage relative to vehicle-treated controls.

### 2.5. Collagen Synthesis

Collagen synthesis was quantified using a previously described collagenase-sensitive [^3^H]proline incorporation assay [36]. This method exploits the susceptibility of collagen to collagenase digestion, allowing differentiation between collagenous and non-collagenous protein synthesis. Cells were pulsed with [^3^H]proline (2 µCi/mL) in culture medium supplemented with ascorbic acid (50 µg/mL) and β-aminopropionitrile (80 µg/mL) for 48 h at 37 °C. Following the 48 h radiolabeling period, cells were washed with ice-cold PBS to remove unincorporated [^3^H]proline and soluble proteins. Cells were then lysed on ice with Tris buffer (0.1 M, pH 7.4) containing NaCl (0.65 M), CaCl_2_ (5.0 mM), *N*-ethylmaleimide (2.5 mM), and bovine serum albumin (100 µg/mL). Cell lysates were divided into two equal aliquots for parallel processing. To measure total protein synthesis, the first aliquot was immediately precipitated by adding an equal volume of ice-cold TCA to achieve a final TCA concentration of 10%, which precipitates all proteins, including collagen. To measure non-collagenous protein synthesis, the second aliquot was incubated with highly purified collagenase for 90 min at 37 °C with gentle agitation to specifically digest collagen into small, acid-soluble peptides. Following digestion, proteins were precipitated with TCA (10%) as described above, leaving collagen-derived peptides in the supernatant. Both TCA-precipitated samples were centrifuged at 14,000× *g* for 10 min at 4 °C to pellet acid-insoluble proteins. Pellets were washed with ice-cold TCA (10%), followed by ice-cold ethanol to remove residual TCA and unincorporated radioactivity, then air-dried to remove residual ethanol. Dried pellets were dissolved in SDS (0.2%)/NaOH (0.2 N) and incubated at 60 °C for 1 h to ensure complete solubilization. Radioactivity was determined by liquid scintillation counting (Tricarb liquid scintillation analyzer, model 2100, Packard, Meriden, CT, USA), and de novo collagen synthesis was calculated as the difference in [^3^H]proline incorporation between the non-digested samples (total protein) and the collagenase-digested samples (non-collagenous protein). Values were normalized for DNA content and expressed as a percentage relative to control conditions.

### 2.6. Cytotoxicity Assay

Cytotoxicity was assessed by monitoring lactate dehydrogenase activity in the culture medium using the CytoTox 96^®^ Non-Radioactive Cytotoxicity Assay, according to the manufacturer’s instructions (Promega Life Sciences, Madison, WI, USA) [37]. Briefly, culture medium overlaying cells was collected, transferred to a 96-well plate, and mixed with an equal volume of CytoTox 96^®^ reagent. The plate was incubated at room temperature for 30 min in the dark. The reaction was terminated by adding the stop solution, and absorbance was measured at 490 nm using a µQuant spectrophotometer (Bio-Tek Instruments, Winooski, VT, USA). Lactate dehydrogenase activity was calculated as a percentage of maximal lactate dehydrogenase activity, which was determined by lysing cells with the provided lysis buffer.

### 2.7. Western Blotting

Cells were rinsed with ice-cold PBS and lysed in Laemmli sample buffer (125 mM Tris, pH 6.8, 12.5% glycerol, 2% SDS, and 0.01% bromophenol blue), supplemented with 5% β-mercaptoethanol. Lysates were heated at 95 °C for 5 min, and equal amounts of protein were separated by SDS-PAGE. Proteins were transferred to nitrocellulose membranes (GE Healthcare, Chicago, IL, USA), and transfer efficiency was confirmed by Ponceau S staining. Membranes were blocked with 5% dry milk in PBS for 60 min at room temperature and then incubated overnight at 4 °C with primary antibodies against GLS1 (1:2000) or β-actin (1:1500). Membranes were then incubated with appropriate horse-radish peroxidase-conjugated antibodies (1:2000) for 60 min at room temperature, and signals were detected using enhanced chemiluminescence substrate (GE Healthcare, Chicago, IL, USA). Immunoreactive bands were acquired using the ChemidocTM Imaging System (Bio-Rad Laboratories, Hercules, CA, USA), and band intensities were calculated using SigmaScan Pro 5 software (Systat Incorporated, Richmond, CA, USA) and normalized to β-actin loading controls [38].

### 2.8. Measurement of Intracellular Glutamate and αKG

Glutamate and αKG levels were measured using enzyme-linked assays. Cells were washed twice with ice-cold PBS, harvested by scraping, and lysed in assay buffer provided with each kit. Lysates were centrifuged at 13,000× *g* for 10 min at 4 °C to remove insoluble material, and supernatants were collected for analysis. Glutamate concentrations were determined using the Glutamate Assay Kit according to the manufacturer’s instructions (Sigma-Aldrich, St. Louis, MO, USA). Samples were incubated with a glutamate enzyme mix and glutamate developer for 30 min at 37 °C in the dark. Absorbance was measured at 450 nm using a uQuant spectrophotometer (Bio-Tek Instruments, Winooski, VT, USA). Lysate αKG levels were quantified using the α-Ketoglutarate Assay Kit. The assay employs a coupled enzyme reaction in which αKG is converted to a detectable product. Samples were processed according to the manufacturer’s instructions, and absorbance or emission was ascertained by spectroscopy. Glutamate and αKG concentrations were calculated from standard curves generated using authentic standards and normalized to total protein content as measured using the Pierce™ BCA Protein Assay (Thermo Fisher Scientific, Waltham, MA, USA). Results are expressed as nmol/mg protein.

### 2.9. Small Interfering RNA (siRNA) Transfection

Gene expression was silenced using siRNA targeting GLS1 (Dharmacon, Lafayette, CO, USA). Cells were transfected with siRNA specific for GLS1 or NT-scrambled siRNA (50 nM) using Lipofectamine.

### 2.10. Statistical Analysis

Data are presented as mean ± SEM. For comparisons between two groups, Student’s unpaired two-tailed *t*-test was employed, while one-way analysis of variance with the Holm–Sidak post hoc test was performed for comparisons among three or more groups. Statistical analyses were performed using SigmaPlot v16.0 (SyStat Software Incorporated, San Jose, CA, USA). Statistical significance was set at *p* < 0.05.

## 3. Results

**Figure 1:**
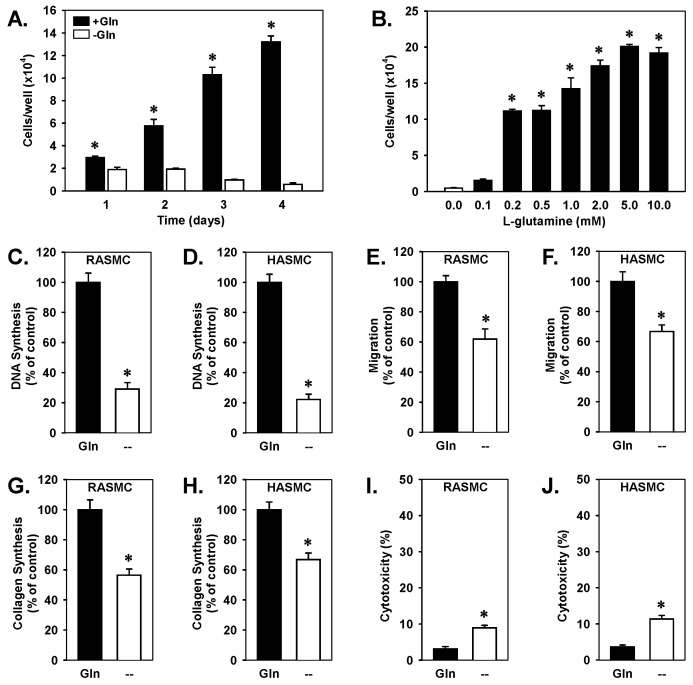
Glutamine (Gln) stimulated proliferation, migration, collagen synthesis, and survival of vascular smooth muscle cells (VSMCs). (**A**) Gln stimulated the proliferation of rat aortic VSMCs in a time-dependent manner. (**B**) Gln stimulated the proliferation of rat aortic VSMCs in a concentration-dependent manner. VSMCs were incubated in the presence or absence of varying concentrations of Gln for four days. (**C**,**D**) Gln deprivation for three days inhibited DNA synthesis in rat and human aortic VSMCs. (**E**,**F**) Gln deprivation for three days inhibited the migration of rat and human aortic VSMCs. (**G**,**H**) Gln deprivation for three days inhibited collagen synthesis in rat and human aortic VSMCs. (**I**,**J**) Gln deprivation for three days reduced the viability of rat and human aortic VSMCs. Results are means ± SEM (*n* = 6). Statistical analysis was performed using analysis of variance with the Holm–Sidak post hoc test (**A**,**B**) or Student’s unpaired two-tailed *t*-test (**C**–**J**). * Statistically significant effect of Gln addition or removal.

Glutamine was essential for the proliferation of rat aortic VSMCs, with no cell growth observed in glutamine-depleted media (Figure 1A). However, supplementation with glutamine induced a significant, concentration-dependent increase in cell proliferation (Figure 1B). DNA synthesis was similarly compromised by glutamine-depletion in both rat and human aortic VSMCs (Figure 1C,D). Furthermore, glutamine removal from culture media blocked VSMC migration in cells derived from both species (Figure 1E,F) and inhibited collagen synthesis (Figure 1G,H). Glutamine depletion also caused a modest but significant increase in cell death in both rat and human VSMCs (Figure 1I,J).

**Figure 2:**
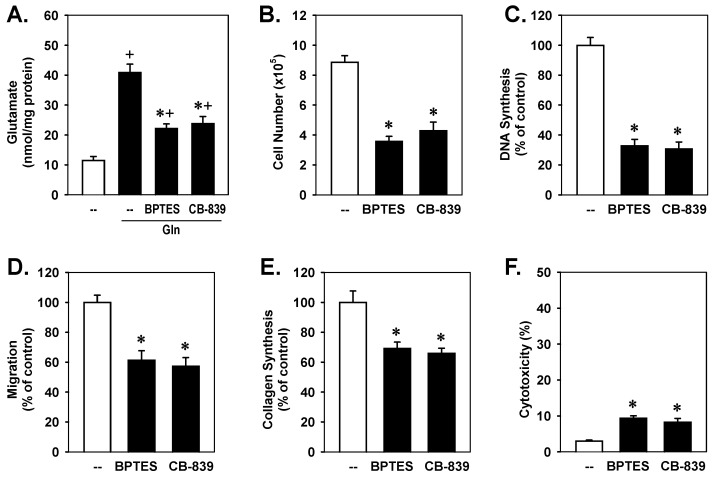
GLS1 inhibition blocked glutamine (Gln)-mediated increases in intracellular glutamate and vascular smooth muscle cell (VSMC) proliferation, migration, collagen synthesis, and survival. (**A**) Gln-mediated increases in intracellular glutamate were inhibited by GLS1 inhibitors, BPTES, and CB-839. (**B**–**F**) BPTES and CB-839 inhibited VSMC proliferation, DNA synthesis, migration, collagen synthesis, and viability. Rat aortic VSMCs were incubated with Gln (2 mM) in the presence or absence of BPTES (20 µM) or CB-839 (20 µM) for 3 days. Results are means ± SEM (*n* = 6). Statistical analysis was performed using analysis of variance with the Holm–Sidak post hoc test. ^+^ Statistically significant effect of Gln. * Statistically significant effect of GLS1 inhibitors.

Since glutamine is largely metabolized by GLS1 [4], we next determined the role of this enzyme in VSMC function. VSMCs grown in glutamine-depleted media exhibited low intracellular glutamate concentrations, while glutamine supplementation resulted in an approximate four-fold increase in intracellular glutamate levels. This spike in glutamate following glutamine supplementation was largely attenuated by treating VSMCs with the GLS1-specific small-molecule inhibitors BPTES and CB-839 (Figure 2A) [39,40], verifying their efficacy in blocking GLS1 activity. The GLS1 inhibitors also blocked proliferation, migration, DNA synthesis, and collagen production in VSMCs grown in glutamine-replete media (Figure 2B–E). In addition, BPTES and CB-839 modestly reduced VSMC viability (Figure 2F).

**Figure 3:**
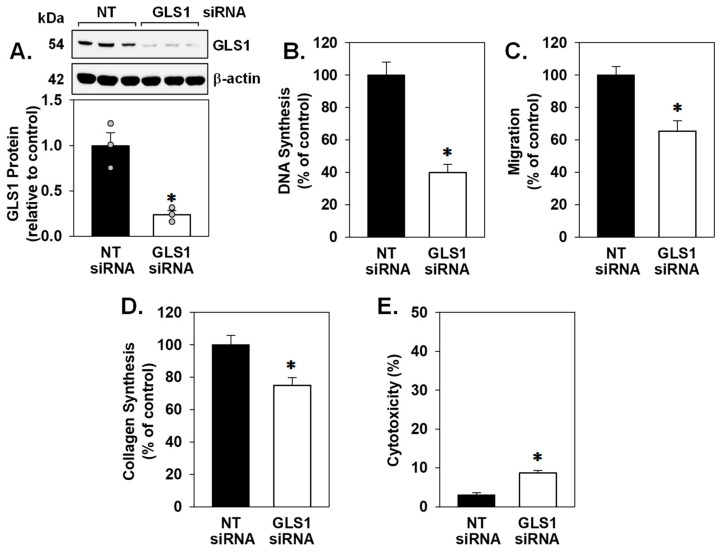
GLS1 knockdown inhibited vascular smooth muscle cell (VSMC) proliferation, migration, collagen synthesis, and survival. (**A**) GLS1 siRNA inhibited GLS1 protein expression. (**B**–**E**) GLS1 siRNA inhibited VSMC proliferation, migration, collagen synthesis, and viability. VSMCs were transfected with GLS1 siRNA (50 nM) or non-targeting (NT) siRNA (50 nM) for three days. Results are means ± SEM (*n* = 3–6). Statistical analysis was performed using Student’s unpaired two-tailed *t*-test. * Statistically significant effect of GLS1 silencing.

To further corroborate a role for GLS1 in modulating VSMC function, we employed an siRNA approach to knock down GLS1 expression. Transfection of VSMCs with GLS1 siRNA decreased the expression of GLS1 protein by approximately 70% (Figure 3A). Moreover, silencing GLS1 expression decreased proliferation, migration, collagen synthesis, and viability of VSMCs cultured in glutamine-containing media (Figure 3B–E).

**Figure 4:**
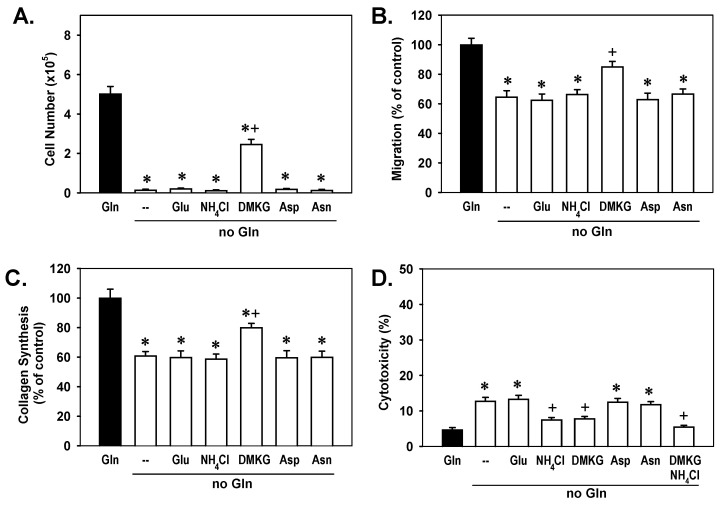
Effect of glutamine (Gln) metabolites on the activation and survival of Gln-deprived vascular smooth muscle cells (VSMCs). (**A**,**B**) α-Ketoglutarate (αKG) restored the proliferation and migration of rat aortic VSMCs. (**C**) αKG restored collagen synthesis in Gln-deprived rat aortic VSMCs. (**D**) αKG and ammonia (NH_3_), given as ammonium chloride (NH_4_Cl), restored the viability of Gln-deprived rat aortic VSMCs. Cells were grown in Gln-replete (2 mM) or -deprived conditions with or without supplementation of glutamate (Glu; 2 mM), NH_4_Cl (2 mM), dimethyl-αKG (DMKG; 2 mM), aspartate (Asp; 100µM), and/or asparagine (Asn; 100 µM) for three days. Results are means ± SEM (*n* = 6). Statistical analysis was performed using analysis of variance with the Holm–Sidak post hoc test. * Statistically significant effect of Gln deprivation. ^+^ Statistically significant effect of DMKG and/or NH_4_Cl supplementation.

To identify which of the glutamine metabolites regulate VSMC function, we supplemented glutamine-deprived cultures with various downstream metabolites. Neither NH_3_, administered as ammonium chloride, nor glutamate rescued proliferation, migration, or collagen formation in glutamine-deprived VSMCs (Figure 4A–C). In contrast, the cell-permeable αKG analog dimethyl-αKG substantial restored VSMC growth, migration, and collagen synthesis. Given that glutamine also contributes to the cellular pools of asparagine and aspartate, we investigated whether these amino acids might mediate glutamine’s effects. However, supplementation with either aspartate or asparagine failed to rescue any of the measured VSMC functions. The addition of dimethyl-αKG also restored cell viability in VSMCs cultured under glutamine-deficient conditions (Figure 4D). Interestingly, ammonium chloride blocked the cytotoxic effect of glutamine withdrawal and enhanced the pro-survival effect of dimethyl-αKG. Conversely, glutamate, aspartate, or asparagine supplementation did not improve viability in glutamine-starved VSMCs.

**Figure 5:**
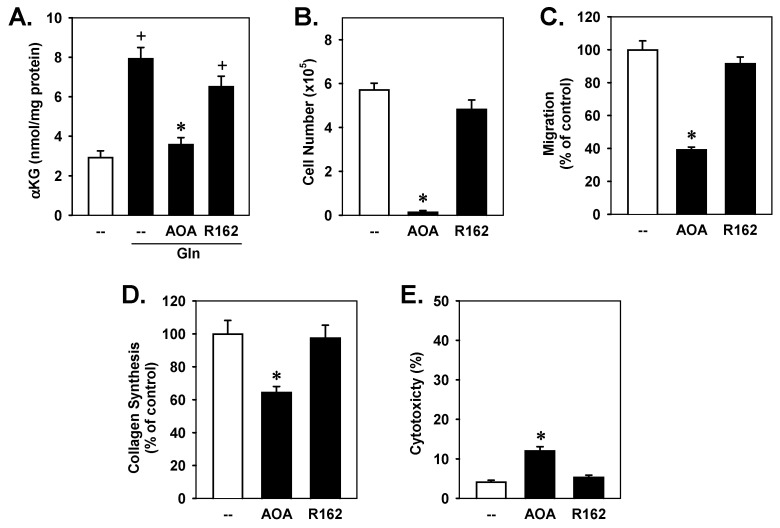
Aminotransferase inhibition blocked glutamine (Gln)-mediated increases in intracellular α-ketoglutarate (αKG) and vascular smooth muscle cell (VSMC) proliferation, migration, collagen synthesis, and survival. (**A**) Gln-mediated increases in intracellular αKG were inhibited by the aminotransferase inhibitor aminooxyacetic acid (AOA) but not by the GLUD1 inhibitor R162. (**B**–**E**) Aminotransferase, but not GLUD1, inhibition blocked VSMC proliferation, migration, collagen synthesis, and viability. Rat aortic VSMCs were incubated with Gln (2 mM) in the presence or absence of AOA (2 mM) or R162 (20 µM) for three days. Results are mean ± SEM (*n* = 5–6). Statistical analysis was performed using analysis of variance with the Holm–Sidak post hoc test. ^+^ Statistically significant effect of Gln. * Statistically significant effect of AOA.

Given the importance of αKG in mediating the actions of glutamine in VSMCs, we investigated the enzymatic pathway by which glutamate is metabolized into αKG. Glutamate can be metabolized to αKG and NH_3_ by GLUD1 or to αKG and non-essential amino acids by aminotransferases [1,4]. To dissect the contribution of these two enzymatic pathways in generating αKG, VSMCs were treated with R162, a specific inhibitor of GLUD1, or AOA, a pan-inhibitor of aminotransferases [41,42]. The administration of glutamine induced a significant rise in the cellular concentration of αKG that was strikingly inhibited by AOA, while R162 only modestly affected αKG levels, indicating a primary role for aminotransferases in mediating αKG production in VSMCs (Figure 5A). Notably, aminotransferase inhibition with AOA significantly inhibited VSMC proliferation, migration, collagen synthesis, and viability, while GLUD1 inhibition with R162 had no effect (Figure 5B–D). Collectively, these findings indicate that aminotransferase-driven αKG production from glutamine controls VSMC function.

## 4. Discussion

**Figure 6:**
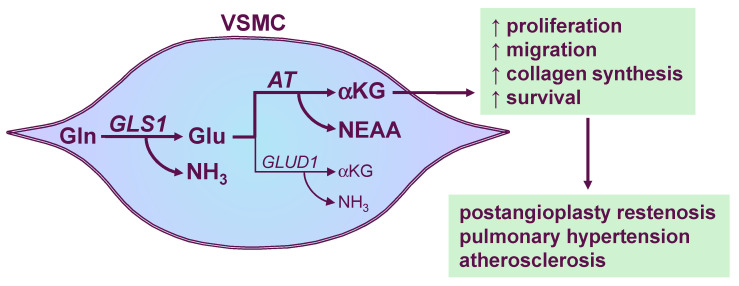
Model for glutamine (Gln) metabolism and its regulatory functions in vascular smooth muscle cells (VSMCs). Gln undergoes mitochondrial glutaminolysis where glutaminase-1 (GLS1) converts it to glutamate (Glu) and ammonia (NH_3_). Glu is primarily catabolized by aminotransferases (AT) to generate alpha-ketoglutarate (αKG) and non-essential amino acids (NEAA), while glutamate dehydrogenase-1 (GLUD1) contributes minor amounts of αKG and NH_3_ in VSMCs. The Gln-αKG metabolic axis is essential for VSMC proliferation, migration, collagen synthesis, and survival. Consequently, therapeutic approaches targeting GLS1 and AT represent a promising strategy for treating fibroproliferative vascular disease, including post-angioplasty restenosis, pulmonary hypertension, and atherosclerosis.

This study establishes glutamine as a critical regulator of VSMC activation and survival. Glutamine stimulates VSMC proliferation, migration, collagen synthesis, and viability in cells derived from both rat and human arteries, revealing conservation of this metabolic pathway across animal species. Mechanistically, glutamine undergoes sequential metabolism by GLS1 and aminotransferases to elevate cellular αKG levels, which serves as the primary mediator of glutamine’s downstream effects on VSMC function (see Figure 6). Supporting this model, pharmacological or molecular inhibition of either GLS1 or aminotransferases abolishes glutamine-dependent VSMC responses. These findings identify the glutamine-αKG metabolic axis as a central driver of VSMC activation and survival, and position GLS1 and aminotransferases as promising therapeutic targets for treating fibroproliferative vascular disease, including post-angioplasty restenosis, pulmonary hypertension, and atherosclerosis.

The current study demonstrates that glutamine promotes both growth and migration of VSMCs, with a critical distinction: VSMC proliferation is strictly dependent on glutamine availability, whereas VSMC migration, although substantially reduced, persists under glutamine-free conditions. Glutamine has long been recognized as an essential nutrient in cell cycle progression, enabling passage through the G1 restriction point and entry into S phase [43,44]. Consistent with this established role, glutamine withdrawal from the culture media severely impaired VSMC growth and DNA synthesis. Both proliferative and migratory responses to glutamine require GLS1 activity, as evidenced by their inhibition using two discrete pharmacological GLS1 inhibitors or by the siRNA-mediated knockdown of GLS1. These findings align with recent studies in rodent-derived VSMCs showing glutamine-dependent proliferation and migration mediated by GLS1 [27,32]. Importantly, we extend these observations by establishing that GLS1 similarly regulates human VSMC growth and motility, suggesting evolutionary conservation of this mechanism and potential translational relevance. Furthermore, we show that glutamine’s effects on VSMCs depend specifically on glutamate catabolism via aminotransferases rather than GLUD1. Treatment with the aminotransferase inhibitor AOA impaired both glutamine-mediated VSMC proliferation and migration, whereas GLUD1 inhibition had no effect. Supporting this pathway specificity, the glutamine-induced increase in intracellular αKG concentration was blocked by AOA but unaffected by the GLUD1 inhibitor R162, establishing aminotransferases as the critical enzymatic route for glutaminolysis in VSMCs. This preferential use of aminotransferases aligns with stable isotope studies identifying glutaminolysis as the primary αKG source in cardiac myofibroblasts, and with findings in mammary epithelial cells showing that proliferating cells catabolizing glutamate via aminotransferases generate both αKG and non-essential amino acids, whereas quiescent cells metabolize glutamate through GLUD1, thereby limiting amino acid production [4,45,46].

The sequential metabolism of glutamine by GLS1 and aminotransferases generates metabolites essential for nucleotide, protein, and lipid synthesis required for cell proliferation and migration. Consequently, glutamine deprivation may restrict VSMC function by limiting these crucial metabolites. Supporting this hypothesis, we found that supplementation with cell-permeable dimethyl-αKG restores both proliferation and migration in glutamine-starved VSMCs. This rescue effect likely operates through multiple mechanisms. First, by replenishing the TCA cycle, αKG fulfills the bioenergetic and biosynthetic demands of proliferating and migrating cells [1,2,3]. Second, αKG directly stimulates lysosomal translocation and activation of mammalian target of rapamycin complex 1 (mTORC1), a master regulator of protein synthesis and cell growth [47]. Third, αKG functions as an agonist for the G-protein coupled receptor GPR99, which has been linked to the proliferation of endothelial cells and may similarly promote VSMC growth [48,49]. Fourth, αKG triggers the activation of signaling pathways and transcriptomic profiles that favor proliferation and migration [50].

Our finding that asparagine fails to rescue proliferation in glutamine-deprived VSMCs contrasts sharply with observations in multiple cancer cell lines, where asparagine supplementation can partially or fully restore growth conditions under glutamine-limited conditions. However, our results align closely with studies in endothelial cells where asparagine alone cannot restore proliferative capacity during glutamine withdrawal, despite being successfully taken up by cells [51,52,53]. This discrepancy between malignant and non-malignant vascular cells suggests cell-type-specific differences in asparagine metabolism, asparagine-dependent metabolic pathways, or the relative importance of asparagine synthesis versus uptake in meeting cellular biosynthetic demands. Supporting this interpretation of cell-type-specific asparagine responses, the inability of asparagine to improve viability in glutamine-deprived VSMCs diverges substantially from studies in tumor cells, where asparagine supplementation suppresses glutamine-withdrawal-induced apoptosis through mechanisms involving the integrated stress and activating transcription factor 4-mediated transcriptional adaptation, specifically via suppression of the proapoptotic transcription factor C/EBP homologous protein [54,55]. This protective effect in cancer cells may reflect fundamental differences in asparagine metabolism and function between malignant and non-malignant cells, including alterations in amino acid transporter expression, differences in the coupling between asparagine availability and nucleotide biosynthesis, or divergent dependencies on asparagine for maintaining protein synthesis rates during nutrient stress [54,56,57,58]. Similarly, supplementation with glutamate or aspartate failed to restore proliferation and migration in glutamine-starved VSMCs, despite these amino acids serving as direct metabolic products of glutamine catabolism and representing potential nitrogen and carbon sources for biosynthetic pathways. This observation parallels findings in cultured human endothelial cells and likely stems from the restricted membrane permeability of these dicarboxylic negatively charged amino acids in vascular cells at physiological pH [9]. Furthermore, NH_3_ supplementation also failed to rescue VSMC function under glutamine deficiency, indicating that NH_3_, a major glutamine-derived metabolite, does not play a critical role in regulating VSMC growth and motility.

We also found that glutamine is essential for maintaining collagen synthesis by VSMCs. Glutamine withdrawal from culture media resulted in a pronounced decline in collagen synthesis that was recapitulated by the silencing of GLS1 and pharmacological inhibition of either GLS1 or aminotransferase, demonstrating that glutaminolysis is critical for VSMC collagen production. These findings align with earlier studies showing that glutamine deprivation reduces collagen gene transcription in cultured mouse VSMCs and human fibroblasts and with more recent studies demonstrating that collagen synthesis by myofibroblasts derived from lung, cardiac, renal, hepatic, or pancreatic stellate cells depends on glutaminase activity [27,45,59,60,61,62,63]. Notably, we found that aminotransferase activity is also required for collagen synthesis, revealing a potentially novel role for these enzymes in vascular fibrosis. Rescue experiments demonstrated that only a cell-permeable analogue of αKG restored collagen synthesis in glutamine depleted conditions, whereas supplementation with amino acids (glutamate, aspartate, and asparagine) or NH_3_ failed to rescue collagen formation. This unique ability of αKG to functionally replace glutamine and restore collagen synthesis has been similarly reported in myofibroblasts [11,45,59,61].

Several mechanisms likely mediate αKG’s contribution to collagen synthesis. Most fundamentally, αKG serves as an obligatory co-substrate for 2-oxoglutarate-dependent dioxygenases, a diverse family of conserved enzymes that catalyze oxidation reactions across a broad range of substrates. Among these, αKG is required by Jumonji C domain-containing lysine demethylases (the major histone demethylases) and ten-eleven translocation hydroxylases involved in DNA demethylation, both of which catalyze the oxidative decarboxylation of αKG. Through these epigenetic modifiers, αKG can promote collagen gene expression by facilitating DNA and histone demethylation [63,64]. Beyond transcriptional regulation, αKG enhances collagen synthesis at the post-transcriptional level by promoting collagen translation through mTORC1 activation and stabilizing collagen structure via proline hydroxylation [59]. Additionally, αKG expands the cellular pool of proline, a critical collagen building block, either by serving directly as a proline precursor or by activating prolidase, which recycles proline from degraded proteins [1,65]. Finally, given the substantial energetic demands of collagen synthesis driven by its large size, unique amino acid composition, extensive post-translational modifications, and complex assembly and secretion processes, αKG may support collagen production by generating sufficient ATP to fuel the biosynthesis of this fibrous structural protein [66,67].

The removal of glutamine from the culture media also increases VSMC death. This adds to earlier work showing that glutamine depletion attenuates endothelial cell survival, suggesting a vital role for this amino acid in preserving vascular cell viability [52]. In addition, as previously reported in mouse aortic VSMCs [27], we observed that inhibition of GLS1 activity or expression diminishes the survival of rat and human aortic VSMCs. Moreover, we found that aminotransferase activity also regulates cell survival, further emphasizing the significance of glutaminolysis in preserving VSMC viability. For the first time, we show that αKG can restore the survival of VSMCs grown in glutamine-deficient media, while other glutamine metabolites such as glutamate, aspartate, and asparagine fail to rescue cell viability. Our finding that glutamine-derived αKG serves as a critical regulator of VSMC survival has also been noted in other cell types, including endothelial cells, cardiac myofibroblasts, and mouse embryonic fibroblasts [45,52,68]. Intriguingly, exogenous NH_3_ supplementation also rescues VSMC viability following the exclusion of glutamine from the culture media. However, aminotransferase inhibition, which depletes αKG but should preserve NH_3_ levels, still triggers VSMC death, demonstrating that endogenous NH_3_ cannot compensate for αKG loss. This discrepancy may reflect that endogenous NH_3_ concentrations remain below the threshold required for cytoprotection. Alternatively, subcellular compartmentalization may restrict mitochondrial-generated NH_3_ to local sites, preventing access to critical survival-regulating compartments that exogenous NH_3_ reaches following bulk supplementation. Further investigation is needed to determine whether NH_3_ concentration, spatial distribution, or both account for the differential effects of endogenous versus exogenous NH_3_ on VSMC survival.

αKG protects against cell death through multiple complementary mechanisms. It directly neutralizes reactive oxygen species such as hydrogen peroxide and peroxynitrite via non-oxidative decarboxylation to yield succinate, CO_2_, and water [69]. Beyond this direct activity, αKG upregulates the expression and activity of numerous antioxidant enzymes, thereby enhancing cellular defenses against oxidative damage [70]. At the mitochondrial level, αKG preserves mitochondrial energy generation, mitochondrial mass, respiratory capacity, and membrane potential, which collectively limit reactive oxygen species production and cytochrome c release, thereby preventing apoptosis [11]. Additionally, αKG promotes mitophagy to selectively eliminate damaged mitochondria [71]. Through its role as an essential cofactor for Jumonji C domain-containing histone demethylases, αKG can activate anti-apoptotic gene expression programs by removing repressive histone methylation marks at specific genomic loci [45]. Moreover, αKG attenuates ferroptosis by upregulating the glutathione peroxidase 4 pathway and suppressing lipid peroxidation [70]. In contrast, NH_3_ inhibits cell death through distinct alternative pathways: it induces heme oxygenase-1 expression with subsequent release of the cytoprotective gas carbon monoxide and stimulates autophagy, which facilitates the recycling of damaged cellular components to sustain energy homeostasis under stress conditions [72,73].

Glutamine metabolism is highly dynamic and responsive to pathological stimuli. In hypoxic tumor cells, GLS1 and GLUD1 expression and activity increase, promoting glutamine-to-αKG flux [74,75]. Concurrently, tumor cells undergo metabolic rewiring from oxidative metabolism to reductive carboxylation, wherein isocitrate dehydrogenase converts αKG to isocitrate and subsequently citrate for fatty acid synthesis, bypassing the conventional αKG dehydrogenase-mediated oxidative pathway to succinate [76]. This adaptation is essential because hypoxia diverts glucose toward lactate production rather than TCA cycle oxidation, necessitating alternative carbon sources for biosynthesis. These metabolic changes enable cancer cells to survive, proliferate, and metastasize within hypoxic microenvironments.

Emerging evidence indicates that hypoxia similarly reprograms glutamine metabolism in non-malignant conditions. Paralleling observations in tumor cells, metabolic profiling reveals that pulmonary tissues from patients with pulmonary arterial hypertension exhibit substantial reductive carboxylation activity that intensifies under hypoxic conditions, supporting the biosynthetic demands of proliferating cells and contributing to pathologic vascular remodeling in pulmonary hypertension [77]. Beyond hypoxia, mechanical arterial injury also activates glutamine metabolism. Wire injury of mouse carotid arteries induces GLS1 expression in neointimal VSMCs, likely driven by growth factor production at sites of vascular damage [27]. Administration of the GLS1 inhibitor CB-838 significantly reduces neointimal area, demonstrating that glutamine metabolism is both active and necessary for the injury response. Similarly, glutamine antagonists or transport inhibitors attenuate neointimal thickening in murine carotid artery ligation models, further establishing the critical role of glutaminolysis in driving neointima formation [27,32,33]. Collectively, these findings demonstrate that pathological conditions prevalent in fibroproliferative vascular diseases, including hypoxia, mechanical injury, and growth factor signaling, converge on activating the glutamine-αKG metabolic axis. This metabolic activation not only supports the proliferative, migratory, and synthetic functions of VSMCs but also suggests that the therapeutic benefit of targeting GLS1 and aminotransferases may be amplified in disease contexts where these pathological stimuli are operative.

This cell-based study provides important new insights into glutamine metabolism and its functional role in VSMCs; however, several questions warrant further investigation. In particular, αKG does not fully restore all the functions of glutamine-depleted VSMCs; it only partially rescues their proliferative and synthetic capacity, indicating that additional metabolites of glutamine modulate VSMC activation. Future studies examining the role of glutamine-derived nucleotides, lipids, glutathione, polyamines, and glucosamines may reveal novel metabolic pathways that control VSMC function [1,2,3,4]. Extending this work to investigate whether glutamine-derived αKG influences VSMC phenotypic switching represents an important next step, particularly given recent evidence that glutaminolysis contributes to VSMC dedifferentiation and that Jumonji C domain-containing lysine demethylases and ten-eleven translocation hydroxylases participate in phenotypic modulation [27,29,78,79,80]. Finally, while GLS1 is known to promote pathological arterial remodeling following vascular injury or disease, the contribution of aminotransferases to this aberrant response remains unclear and represents a potentially valuable therapeutic target [6,20,24,25,26,27].

## 5. Conclusions

This study establishes glutamine-derived αKG as a central metabolic regulator of VSMC proliferation, migration, collagen synthesis, and survival. We demonstrate that the glutaminolytic enzymes GLS1 and aminotransferases are essential drivers of αKG synthesis in VSMCs, with their inhibition potently suppressing VSMC activation and survival. Critically, we reveal that glutamine’s effects depend specifically on aminotransferase-mediated glutamate catabolism rather than GLUD1 activity, uncovering a distinct metabolic route that distinguishes activated VSMCs from their quiescent counterparts. This finding establishes aminotransferases as a previously underappreciated therapeutic target in vascular disease.

The functional significance of this pathway is further underscored by our rescue experiments, which reveal important metabolic complexity: while αKG partially restores multiple VSMC functions under glutamine deprivation, the incomplete rescue demonstrates that additional glutamine-derived metabolites contribute to VSMC activation. This observation highlights the multifaceted nature of glutamine metabolism in VSMC pathobiology and suggests that combinatorial therapeutic approaches targeting multiple nodes within this pathway may prove more effective than single-target strategies. Collectively, this work identifies the glutamine-αKG axis as a critical metabolic vulnerability in activated VSMCs and provides compelling rationale for developing metabolism-targeted therapies that simultaneously inhibit GLS1 and aminotransferases to prevent or treat fibroproliferative vascular disease.

## Data Availability

The original contributions presented in this study are included in the article. Further inquiries can be directed to the corresponding author.
